# High Temperature Continuous Flow Syntheses of Iron Oxide Nanoflowers Using the Polyol Route in a Multi-Parametric Millifluidic Device

**DOI:** 10.3390/nano12010119

**Published:** 2021-12-30

**Authors:** Enzo Bertuit, Sophie Neveu, Ali Abou-Hassan

**Affiliations:** Sorbonne Université, CNRS, Physico-Chimie des Electrolytes et Nanosystèmes InterfaciauX (PHENIX), F-75005 Paris, France; enzo.bertuit@sorbonne-universite.fr (E.B.); sophie.neveu@sorbonne-universite.fr (S.N.)

**Keywords:** millifluidic device, continuous-flow syntheses, polyol route, iron oxide, magnetic nanoflowers, nanoclusters

## Abstract

One of the most versatile routes for the elaboration of nanomaterials in materials science, including the synthesis of magnetic iron oxide nanoclusters, is the high-temperature polyol process. However, despite its versatility, this process still lacks reproducibility and scale-up, in addition to the low yield obtained in final materials. In this work, we demonstrate a home-made multiparametric continuous flow millifluidic system that can operate at high temperatures (up to 400 °C). After optimization, we validate its potential for the production of nanomaterials using the polyol route at 220 °C by elaborating ferrite iron oxide nanoclusters called nanoflowers (CoFe_2_O_4_, Fe_3_O_4_, MnFe_2_O_4_) with well-controlled nanostructure and composition, which are highly demanded due to their physical properties. Moreover, we demonstrate that by using such a continuous process, the chemical yield and reproducibility of the nanoflower synthesis are strongly improved as well as the possibility to produce these nanomaterials on a large scale with quantities up to 45 g per day.

## 1. Introduction

In many synthesis fields, controlling the reproducibility and the yield of chemical reactions is of primary importance. With such objectives, new types of reactors have been developed and studied over the recent decades. Decreasing the dimensions of the chemical reactor was found to be a solution to improve mass and heat transfer of the reactive media, minimizing the undesired gradients often encountered in bulk [1,2,3,4,5]. Despite their small size, high production capacities can be reached in such reactors when chemical syntheses are performed under continuous-flow conditions, often called milli/micro/nano-fluidic syntheses. Moreover, it has been shown that the yield and reproducibility of the synthesis are greatly enhanced in such reactors, again due to their small dimensions. In the field of nanomaterials synthesis, a large number of microfluidic reactors have been developed to carry chemical synthesis in a greener and safer way starting from ambient temperature to high-temperature and high-pressure supercritical systems allowing the production of a large panel of high-quality nanoparticles (NPs) [6,7,8,9,10,11,12,13,14,15,16].

In the particular case of iron oxide syntheses, different strategies have already been developed, but they involve the design of complex reactors (micromixers, multiphasic reactors) [17,18], the use of special methodologies (gas slugs, water-in-oil droplets) [8,19,20] or are only suitable for temperatures below 100 °C [10,21,22,23,24] which strongly limits the possibility to obtain highly crystalline NPs. However, such reactors could advance the field of high-temperature materials science syntheses, for which heat transfer and temperature homogeneity govern the physico-chemical features of the as-synthetized nanomaterials. Despite the possible advantages of such reactors, the use of high-temperature devices for the continuous-flow elaboration of iron oxide NPs is still scarce in the literature. Indeed, only ultra-small iron oxide NPs (4 nm to 8 nm nanospheres) obtained by thermal decomposition of acetylacetonate precursors (from 180 °C to 300 °C) are reported [7,25,26,27].

Multi-core nanoclusters, also called iron oxide nanoflowers (NFs), are multi-core NPs formed by the assembly of several iron oxide cores. Among these nanoclusters, magnetic NFs have received lot of attention recently due to their promising magnetic properties [28,29] which paved the way for many applications [30,31,32,33,34,35,36,37] including as efficient magnetic resonance imaging (MRI) contrast agents [38], as nano-heaters in magnetic hyperthermia (MHT) [39,40] and as shown more recently for photothermal therapy (PTT) [40,41,42]. To date, one of the most versatile routes to produce such nanostructures in bulk is the polyol route (high temperatures > 200 °C) where the solvents act both as reducing agents and stabilizers [43]. However, despite the versatility [44,45] of such a process for obtaining NPs with optimized features, it still suffers from a lack of reproducibility in addition to a low yield in final nanomaterials, limiting the scaling-up [46] and subsequently the transposition to different applications. 

In this work, we describe for the first time the continuous flow production of magnetic iron oxide ferrite nanoflowers (CoFe_2_O_4_, Fe_3_O_4_, MnFe_2_O_4_) by using the polyol route and a home-made multi-parametric millifluidic device which enables continuous-flow syntheses at high temperature (up to 400 °C) with great production capacities. We demonstrate that the setup allows a good control over experimental synthesis parameters such as temperature and pressure but also the final features of the NPs including their size, shape and chemical composition. We show also that both the chemical yield and reproducibility are strongly enhanced when compared to polyol synthesis performed in classical reactors. Moreover, the millifluidic system developed in this work has been found to allow very high production capacities and large-scale synthesis (up to 45 g per day) as flow rates can be increased up to 10 mL∙min^−1^.

## 2. Materials and Methods

### 2.1. Chemicals

N-methyldiethanolamine (NMDEA, >99%), diethylene glycol (DEG, >99%), iron(III) chloride hexahydrate (FeCl_3_∙6H_2_O, 99%), iron(II) chloride tetrahydrate (FeCl_2_∙4H_2_O, 99%), cobalt(II) chloride hexahydrate (CoCl_2_∙6H_2_O, 99%) and manganese(II) chloride tetrahydrate (MnCl_2_∙4H_2_O, 99%) are purchased from Merck (Darmstadt, Germany). Sodium hydroxide pellets (NaOH, 99%), hydrochloric acid (HCl, 37%), ethanol (96%), nitric acid (HNO_3_, 68%), acetone (>99%), and diethyl ether (Et_2_O, 100%) are purchased from VWR International (Rosny-sous-Bois, France). All chemicals are used without further purification.

### 2.2. Equipment

Analytical HPLC pump ECP2000 is purchased from ECOM (Prague, Czech Republic). Stainless steel millifluidic channels (inner diameter: 0.040’’, outer diameter: 1/16’’) are purchased from Cluzeau Info Lab (Sainte-Foy-la-Grande, France). Proportional-integral-differential (PID) controllers (77 mm × 35 mm, 220V, −200 to 600 °C), Pt100 temperature probes, heating cartridges (16 mm × 200 mm, 1 kW, 220 V), electrical fuses (16 A and 64 A), on/off switching buttons and electrical cables (1.3 mm^2^ and 2.1 mm^2^) are from Radio Spare Pro™ (Beauvais, France).

### 2.3. Preparation of the Reactive Media

#### 2.3.1. Reactive Media with Various [Co + Fe] Concentrations

FeCl_3_∙6H_2_O and CoCl_2_∙6H_2_O are dissolved in a mixture of DEG (40 mL) and NMDEA (40 mL). At the same time, NaOH pellets are grinded and dissolved overnight in a mixture of DEG (20 mL) and NMDEA (20 mL). The two solutions are mixed by magnetic stirring for 1 h. A volume of 500 mL of ultra-pure water is added and the reactive media is stirred for another 30 min to obtain a ready-to-use reactive media. To respect constant ratios of [Co]/[Fe] and [Co + Fe]/[NaOH], the following quantities are used: 1.08 g of iron(III) salt (4 mmol), 0.48 g of cobalt(II) salt (2 mmol) and 0.64 g of NaOH (for syntheses at [Co + Fe] = 50 mM); 0.36 g of iron(III) salt (1.33 mmol), 0.16 g of cobalt(II) salt (0.67 mmol) and 0.21 g of NaOH (for syntheses at [Co + Fe] = 17 mM); 0.11 g of iron(III) salt (0.4 mmol), 0.05 g of cobalt(II) salt (0.2 mmol) and 0.064 g of NaOH (for syntheses at [Co + Fe] = 5 mM).

#### 2.3.2. Reactive Media with [M + Fe] = 50 Mm (M = Co, Fe, Mn)

The same procedure described previously is followed but using different amounts of metallic salts. To respect a stochiometric ratio of [M]/[Fe^III^] = 0.5 (M = Co, Fe), the following quantities are used: 1.08 g of iron(III) salt (4 mmol), 0.48 g of chloride(II) salt (2 mmol) and 0.64 g of NaOH (for cobalt ferrite syntheses); 1.08 g of iron(III) salt (4 mmol), 0.39 g of iron(II) salt (2 mmol) and 0.64 g of NaOH (for magnetite syntheses). To respect an off-stochiometric ratio of [Mn]/[Fe^III^] = 0.25, the following quantities are used: 1.08 g of iron(III) salt (4 mmol), 0.20 g of manganese(II) salt (1 mmol) and 0.64 g of NaOH (for manganese ferrite syntheses).

#### 2.3.3. Washing Steps

The as-obtained black suspensions (final crude products) are diluted in ethanol to be magnetically separated and washed as follow: one time in diluted HNO_3_ (10%) for ten minutes, two times in acetone and two times in Et_2_O. The black solid is then re-dispersed in a minimum of ultra-pure water to obtain a stable aqueous ferrofluid. Nitrogen is flushed inside the solution to prevent from further oxidation by dissolved oxygen and is conserved in a sealed vial.

### 2.4. Synthesis Procedure

The millifluidic system is filled with DEG using a flow rate of 5 mL∙min^−1^ during 4 min. The PID control boxes are then set to a temperature of 220 °C and the flow rate is decreased to 1 mL∙min^−1^. Once the thermal equilibrium is reached (about 5 min), the reactive media is used as the inlet solution and is injected at the desired flow rate. After a time of *t* = 1.5 τ_R_, the collecting flask is replaced by a new one to collect only the products of the reaction (without collecting the initial DEG or eventual impurities). Once a sufficient volume of crude product is obtained, the collecting flask is replaced by another one. The PID control boxes are set to 20 °C and a DEG flow of 2 mL∙min^−1^ is injected to clean the inside of the millifluidic channel while the temperature decreases. Once the system reaches ambient temperature, 200 mL of water are injected at a high flow rate of 10 mL∙min^−1^ to remove any remaining impurities. Finally, the HPLC pump, each PID control box and the general electrical control box are switched off.

### 2.5. Characterizations

The morphology of the NPs is imaged using a JEOL-1011 (JEOL, Croissy-sur-Seine, France) transmission electron microscope operating at 100 kV. Size distributions are determined thanks to Image J software by measuring manually 300 NPs on at least three different images. The resulting histograms are modelized by a log-normal (Equation (1)) law using Igor Pro 7 software to determine the mean physical diameter (*d*_0_) and the polydispersity (*σ*) of each sample.

(1) Pd=12π·d·σexp−lndd022·σ2

The cobalt, total iron and manganese concentrations of NPs suspensions are measured by atomic absorption spectroscopy (AAS, PinAAcle 500, Perkin Elmer) by degrading the samples in concentrated HCl (37%) before a dilution in HNO_3_ (2%). X-ray absorption spectroscopy at Fe K-edge is performed at Synchtoron SOLEIL on the ROCK line in 1 mm diameter glass capillary, using a Si(111) monochromator. UV-Visible spectra are recorded at room temperature in a 1 cm quartz cuvette using an Avantes spectrophotometric set-up composed of an AvaLight-DHc lamp connected by optical fibers to a StarLine AvaSpec UV-Visible detector. Samples are diluted in analytical grade ethanol in order not to saturate the spectrophotometer and spectra are normalized by the dilution factor. The ultracentrifugation of crude products is performed using a Beckman LC-70 Ultracentrifuge operating at 35,000 rotations per minute. ^1^H NMR spectra are recorded at ambient temperature using a Bruker spectrometer operating at 300 MHz.

## 3. Results and Discussion

### 3.1. Special Strengths of the Multi-Parametric Millifluidic Device

**Figure 1:**
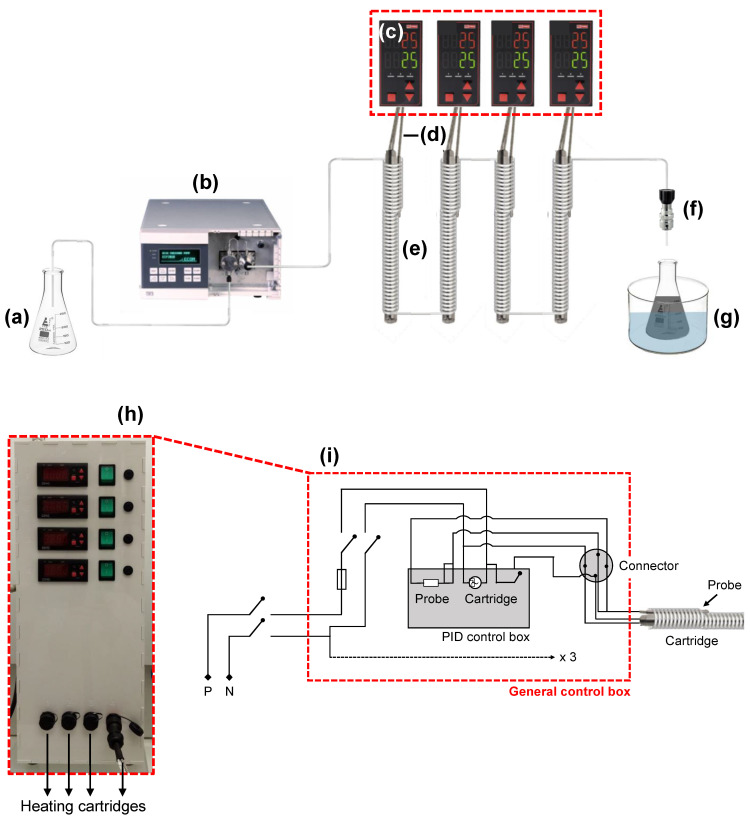
Global description of the multi-parametric millifluidic device. (**a**–**g**) Schematic representation of the different elements composing the system with (**a**) the initial reactive media, (**b**) the HPLC pump, (**c**) the PID control boxes, (**d**) the temperature probes, (**e**) the heating cartridges, (**f**) the back-pressure regulator and (**g**) the collecting flask. (**h**) Photograph of the general control box including all electrical components (red dashed line, Figure 1c). (**i**) Scheme of the electrical connections inside the general control box.

A schematic representation of the multi-parametric and high temperature millifluidic device is shown on Figure 1a–g. The initial reactive media (Figure 1a) is injected into the millifluidic stainless-steel reactor thanks to an analytical high-performance liquid chromatography (HPLC) pump (Figure 1b) operating between 0.1 mL∙min^−1^ and 10 mL∙min^−1^. The temperature of the system is controlled by proportional-integral-differential (PID) control boxes (Figure 1c) connected to both temperature probes (Figure 1d) and heating cartridges (Figure 1e). A back-pressure regulator (BPR) is installed at the outlet of the millifluidic channel (Figure 1f), and the final products of the reaction are collected in a vial refrigerated by a water bath (Figure 1g). The nature of the synthetized products can be easily tuned by modifying the composition of the initial reactive media. The reaction time (or residence time, *τ_R_*) can be controlled with a high precision thanks to the HPLC pump. Such pumps are rarely used [47] even though they present a pressure retro-control so that the flow rate consign remains constant over the whole time of the experiment even if clogging phenomena happen, as opposed to commonly used syringe drivers operating at constant pressure. The reaction temperature is also tunable between ambient temperature and up to 400 °C thanks to the use of high-power heating cartridges (electrical resistances of 1000 W) which efficiently produce heat by Joule effect. A precise control over the reaction temperature is ensured by the PID control boxes, allowing a very good stability of the temperature consign due to a high rate on/off control process of 5 s. The BPR enables modification and control of the reaction pressure (as in microwaves [48] or solvothermal reactors) by maintaining a constant pressure between the inlet and the outlet. Such a control can be very useful to work at temperatures above the ambient temperature boiling point of the used solvents. Finally, as heating cartridges are connected in series, the total length (*L*) of the stainless steel millifluidic channel can be easily increased by adding more cartridges. It thus makes possible a simple and large scaling-up of our system as extremely long channels can be used if high injection flow rates are desired. To ensure the safety of the above-described system in an operational environment, a general electrical control box was designed to include and protect all the electrical components represented on Figure 1c. Figure 1h shows a photograph of the general control box and Figure 1i describes the electrical scheme of the system. Briefly, the whole box is power supplied by a general electrical alimentation equipped with an on/off button secured by a 64 A fuse. Each PID box is controlled by an individual on/off button equipped with a security fuse of 16 A and is linked to a 4 brooches connector allowing a safe and easy plugging of each heating cartridge. 

### 3.2. An Easy and Highly Precise Control of Temperature Conditions

**Figure 2:**
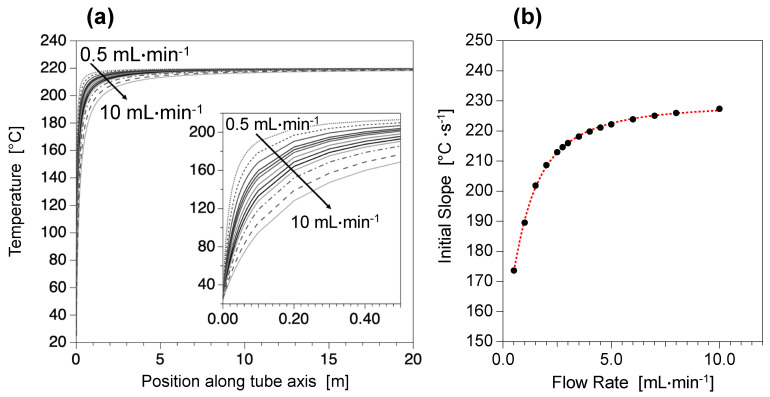
Simulations of heating conditions inside the stainless steel millifluidic reactor for different flow rates showing (**a**) the temperature elevations as a function of the position along tube axis and (**b**) the initial slopes at the early stages of heating. Calculations were performed for increasing flow rates of 0.5 mL∙min^−1^; 1 mL∙min^−1^; 2 mL∙min^−1^; 2.5 mL∙min^−1^; 2.75 mL∙min^−1^; 3 mL∙min^−1^; 3.5 mL∙min^−1^; 4 mL∙min^−1^; 4.5 mL∙min^−1^; 5 mL∙min^−1^; 6 mL∙min^−1^; 8 mL∙min^−1^ and 10 mL∙min^−1^.

Simulations of the temperature increase of the reactive media inside the stainless-steel millifluidic channel are presented on Figure 2. Analytical simulations are performed for different flow rates ranging from 0.5 mL∙min^−1^ to 10 mL∙min^−1^, in the specific case of a volumetric mixture of diethylene glycol (DEG) and N-methyldiethanolamine (NMDEA) which are the most commonly used polyol solvents for magnetic nanoflower synthesis. Assuming that the thickness of the stainless-steel tube (300 mm) permits a perfectly efficient heat conduction, the temperature of the reactive media inside the millifluidic channel at a given position along tube axis can be calculated by Equation 2 [49].

(2) $$\frac{T\left(z\right)-T_{0}}{T_{\mathrm{HC}}-T\left(z\right)}=\frac{\pi \cdot D_{in}}{m_{Q}\cdot C_{p}}\cdot h\cdot z$$

where *T*(z) is the temperature in °C at the position z (in meters) along tube axis, T_0_ is the temperature in °C of the reactor before starting the heating, T_HC_ is the consign temperature in °C of the heating cartridges (fixed at 220 °C here), D_in_ is the tube inner diameter in meters, m_Q_ is the mass flow rate in kg∙s^−1^, C_p_ is the specific heat of the mixture in J∙kg^−1^∙°C^−1^ (herein approximated to the one of DEG only), and *h* is the heat transfer coefficient of the system in W∙m^−2^∙°C^−1^ (see Equations S1–S6 and Figure S1 for more details). Figure 2a evidences that the thermal equilibrium around 220 °C is reached very fast. Indeed, whatever the flow rate is, the temperature stabilizes around 220 °C approximately after 10 sec. The proportion of the tube length necessary to reach such an equilibrium (*L*_220 °C_/*L*_TOT_) is inferior to 10%, which means that temperature stability is ensured on at least 90% of the total length of the reactor. Such a control is of primary importance for high temperature inorganic chemical reactions for which the temperature stability has been proved to control physico-chemical features of the as-synthetized NPs (such as shape, size and crystallinity). Figure 2b shows the variation of the temperature slopes extracted from the analytical simulation in Figure 2a at the early stages of heating (0 < L < 20 cm) as a function of the flow rate. It can be seen that extremely fast heating ramps of 150–250 °C∙s^−1^ can be reached, while standard values in classical reactors are less than 0.2 °C∙s^−1^ [39]. Moreover, Figure 2b evidences that the heating rate depends exponentially on the flow rate, so the heating speed values can be tuned by a simple change of the flow rate. Conversely, Figure 2b shows that for flow rates above 4 mL∙min^−1^, the initial slope becomes almost constant, and elevated flow rates can be used with no significant impact on the heating conditions, making possible a large-scale production of NPs using such high flow rates.

### 3.3. Optimization of the Synthesis Parameters for Ferrite Nanoflowers Production

Cobalt ferrite has been chosen as a model compound for optimization of the microfluidic setup since Co(II) is more stable than Fe(II) toward oxidation, especially at high temperatures, consequently requiring less precautions for manipulation. The effects of several synthesis parameters including the initial concentrations of the metal salt precursors in the mixture and the temperature and pressure of the reactor are investigated. The values of the temperature and pressure are chosen below the boiling point of the solvent, consequently remaining in the liquid phase without reaching the supercritical state. 

**Figure 3:**
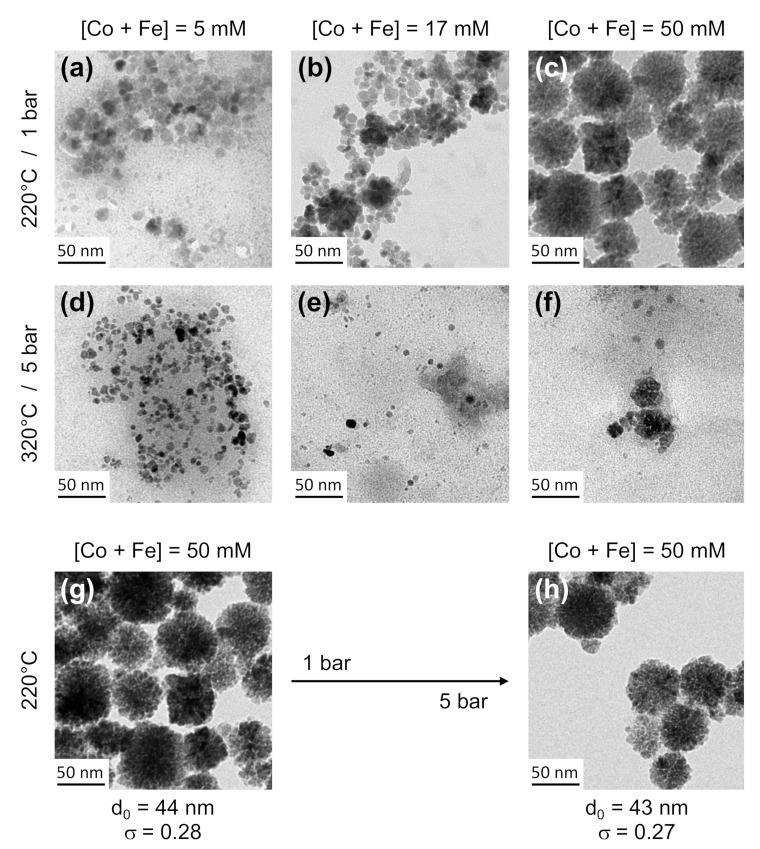
Representative TEM micrographs of CoFe_2_O_4_ nanoflowers obtained for a given residence time of 40 min using different initial precursor concentrations, temperatures and pressures. Approximate diameters are (**a**) 19 nm, (**b**) 21 nm, (**c**) 44 nm), (**d**) 9 nm, (**e**) 10 nm and (**f**) 25 nm. See Figure S2 for size-distribution histograms associated to (**g**) and (**h**) micrographs. All these TEM grids are observed using the crude reactive media before the washing steps to prevent from eventual effects due to the washings.

Figure 3 shows representative TEM micrographs of the final cobalt ferrite NPs obtained by varying the total concentration of Co(II) and Fe(III) in the mixture (5 mM < [Co + Fe] < 50 mM), while maintaining the molar ratio Co(II)/Fe(III) = 0.5 and NaOH/(Co + Fe) = 2.7 with a constant residence time of about 40 min (Q = 0.4 mL∙min^−1^). The first line (Figure 3a–c) shows the effects of increasing the initial concentration for a holding temperature of 220 °C and a pressure of 1 bar while the second line (Figure 3d–f) show results obtained at 320 °C and 5 bars. In both cases, higher concentrations yield larger nanostructures (between 19 nm and 44 nm at 220 °C, between 10 nm and 25 nm at 320 °C) so that growth by aggregation is hindered for low concentrations while it seems possible for a concentration of about 50 mM. However, even at such a concentration, a higher temperature of 320 °C and a pressure of 5 bars results in smaller and poorly defined NFs (Figure 3f). All these qualitative observations evidence that low concentrations and high temperatures have adverse effects over growth of NFs which requires oriented aggregation and attachment. Finally, the optimal parameters for the obtention of well-defined NFs inside the millifluidic device are an initial total concentration in precursors of 50 mM, a temperature of 220 °C and a pressure of 1 bar, in good agreement with the experimental conditions used in classical bulk syntheses performed at atmospheric pressure [39,43,44,45]. If all parameters are maintained constant and a pressure of 5 bars is applied, no effect is evidenced on the resulting NFs (Figure 3h) since they present the same morphology of those obtained at 1 bar (Figure 3g) with an almost final identical size of around 44 nm and neglectable variations of *d*_0_ and *σ* (∆*d*_0_ = 2% and ∆*σ* = 3%). These results evidence that the synthesis can be carried out at 5 bars without affecting the quality of the obtained NFs, which is a more suitable pressure for HPLC pump efficiency and precision. Later in this work, a BPR is used to apply a pressure of 5 bars between the inlet and the outlet.

**Figure 4:**
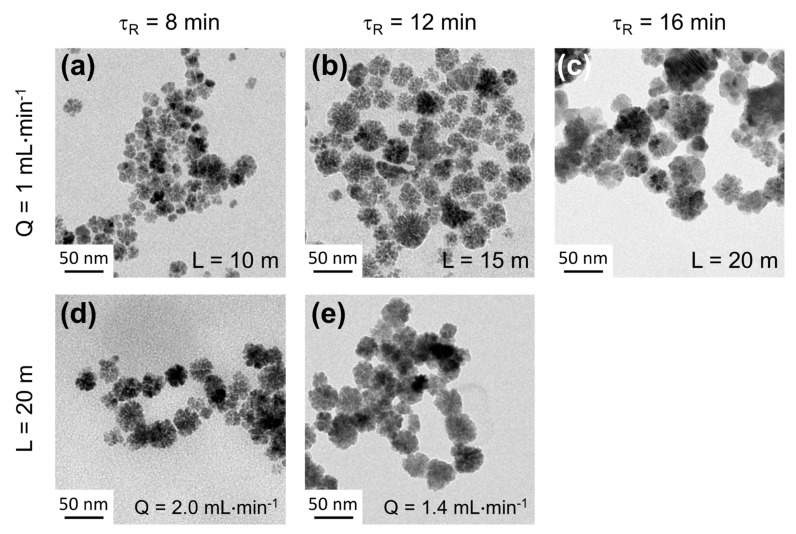
Representative TEM micrographs of Fe_3_O_4_ nanoflowers obtained for different residence times by varying (**a**–**c**) the total length of the channel for a given flow rate of 1 mL∙min^−1^ of (**d**–**e**) by varying the flow rate for a given total length of 20 meters. Values of mean diameters and polydispersity of each sample are (**a**) 33.9 nm, σ = 0.23; (**b**) 35.1 nm, σ = 0.21; (**c**) 36.2 nm, σ = 0.24; (**d**) 33.7 nm, σ = 0.24; (**e**) 35.2 nm, σ = 0.22. All size-distribution histograms are presented in Figure S3.

By assuming that these optimized conditions can be applied to other types of ferrites, for all described experiments in this manuscript, a total concentration of 50 mM in metal salts precursors in the mixture, a ratio R_0_ = [M^2+^]_0_/[Fe^3+^]_0_ = 0.5 (M^2+^ = Co^2+^, Fe^2+^, Mn^2+^), a holding temperature of 220 °C and a pressure of 5 bars are used. Figure 4 shows representative TEM micrographs of magnetite (M^2+^ = Fe^2+^) NFs obtained for different residence times (*τ_R_*) of 8 min, 12 min and 16 min. Such residence times are obtained in two different ways, either by varying the channel length *L* for a constant flow rate *Q* (*τ_R_* = f (*L*), Figure 4a–c) or by varying the flow rate *Q* for a constant channel length L (τ_R_ = f(*Q*); Figure 4d–e). Figure 4a–c shows that slightly bigger NFs are obtained for longer residence times with a size changing from 33 nm to 36 nm with a constant polydispersity of <σ> = 0.22 ± 0.2. Thus, for a given flow rate, the control over polydispersity of the as-synthetized NPs is around 10%. When comparing NFs obtained for identical residence time with different flow rates (Figure 4a,d or Figure 4b,e), values of mean diameter are found to be almost identical with minor changes calculated around 0.5%. Polydispersity values are found to differ from less than 5%, which is less than the previously determined error of 10% for a given flow rate. As a result, flow rate modifications seem to have a very small impact on the size distribution of the as-synthetized NPs. As a consequence, high throughput production and scaling up can be obtained using high flow rates and longer reactor length. 

### 3.4. A Millifluidic Device That Offers a Very Good Control over NPs Physico-Chemical Features

**Figure 5:**
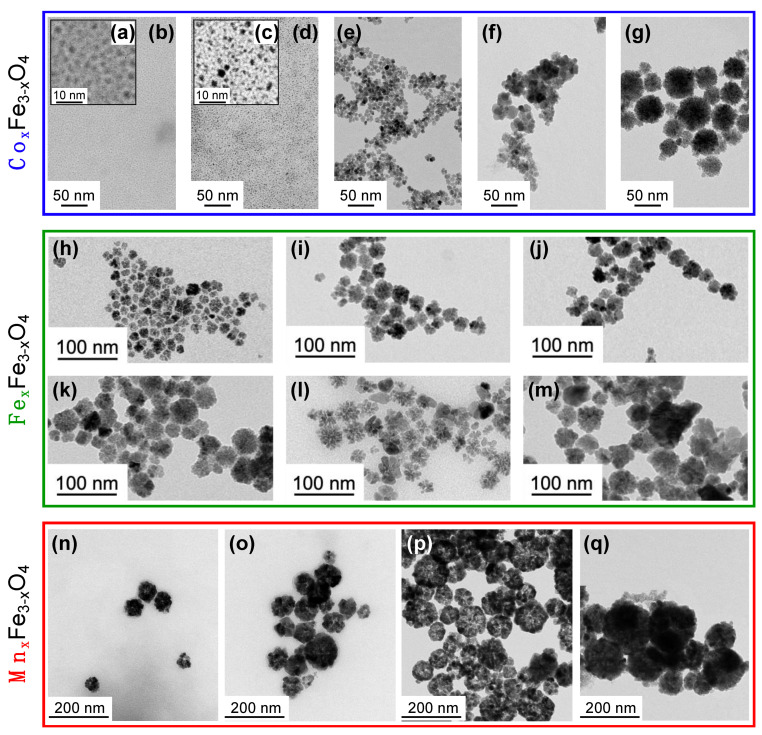
Representative TEM micrographs of nanoflowers obtained using a total tube length of 20 m (4 cartridges) for different initial chemical composition (blue: cobalt; green: iron; red: manganese) and different residence times of (**a**,**b**) 8 min, (**c**,**d**) 16 min, (**e**) 27 min, (**f**) 40 min, (**g**) 35 min, (**h**) 5.3 min, (**i**) 5.8 min, (**j**) 6.4 min, (**k**) 8 min, (**l**) 11 min, (**m**) 16 min, (**n**) 2 min, (**o**) 4 min, (**p**) 8 min and (**q**) 16 min. All size-distribution histograms are presented in Figure S4.

In order to check the potential of our millifluidic system in elaborating different ferrite NPs with controllable physico-chemical features (size, shape and chemical composition, which control the properties of the NPs) [50,51,52,53], three different types of ferrites are synthetized. Syntheses are carried out using previously optimized parameters, i.e., an initial total concentration of precursors of 50 mM, a temperature of 220 °C, a pressure of 5 bars and a reactor length of 20 m. Cobalt ferrite (Co*_x_*Fe_3−*x*_O_4_), magnetite (Fe*_x_*Fe_3−*x*_O_4_) and manganese ferrite (Mn*_x_*Fe_3−*x*_O_4_) are studied for residence times ranging from 8–40 min (Figure 5a–g), 5–16 min (Figure 5h–m) and 2–16 min (Figure 5n–q), respectively.

Figure 5 evidences a general tendency to obtain larger nanostructures for higher residence times, which shows that the size of the as-synthetized NPs can simply be tuned by changing the flow rate. Such a control is of great importance as precise size ranges can be required depending on the desired application for the nanomaterial. The mean diameter and polydispersity of each sample are listed in Table 1. In the case of cobalt ferrite, for a residence time of 8 min and 16 min (Figure 5a–d), ultra-small NPs (around 2 nm) are only observed by comparison to magnetite and manganese ferrite for which even shorter residence times of 5.3 min (Figure 5h) and 2 min (Figure 5h) already show the formation of flower-like structures of 20 nm and 60 nm, respectively. Similar NF organization is obtained for cobalt ferrite but only at longer residence times of about 40 min (Figure 5g) where NFs of 44 nm can be clearly observed. We attribute such differences to a disparity in the kinetics of cobalt ferrite formation when compared to magnetite and manganese ferrite, which is investigated further in the next section. Interestingly, by using the millifluidic setup and by varying the residence times, we can evidence different steps in the formation of cobalt ferrite NFs including the formation of nuclei (Figure 5a–d) and their growth (Figure 5e), followed by their attachment and aggregation into final NFs (Figure 5g). Moreover, our results demonstrate that the optimized parameters determined on cobalt ferrite studies are also well suited for the obtention of well-defined NFs of magnetite or manganese ferrite. These results are thus very promising for the elaboration of other type of magnetic ferrite NFs by polyol route using our millifluidic device.

It is well established that the magnetic properties of ferrite NFs also rely on their chemical composition [54]. As the proportion of M^2+^ and Fe^3+^ in the spinel structure of ferrite affects their magnetic responses, controlling the M^2+^/Fe^3+^ ratio is crucial. The chemical composition of the as-synthetized samples presented in Figure 5 (see Figure S5, Tables S1 and S2 for details) are measured by atomic absorption spectroscopy (AAS) or X-ray absorption near edge spectroscopy (XANES) [55,56,57,58,59,60]. In the case of cobalt ferrite and magnetite, an initial ratio *R*_0_ = [M^2+^]_0_/[Fe^3+^]_0_ = 0.5 is introduced in the reactive media, while *R*_0_ = 0.25 is chosen in the case of manganese ferrite. A comparison of *R*_0_ and composition of the final synthetized products (R_F_ values) is given in Table 1. The results clearly demonstrate a very good correlation between the chemical composition of the reactive media and the stoichiometry of the final products with a mean deviation to ideality of <(*R*_0_ − *R_F_*)/*R*_0_> = 6%. While polyol syntheses in classical round-flask reactors show less incorporation of M^2+^ cations in the final nanostructure (*R_F_* < *R*_0_) [61], polyol millifluidic synthesis permits a better control over the chemical composition and incorporation of divalent ions in the final NPs. These findings may be explained by the efficient thermal transfer due to the small dimensions of the reactors leading to a very good thermal homogeneity of the reactive media inside the millifluidic reactor that strongly impacts the reactivity of the chemical species such as their decomposition.

### 3.5. Towards the Comprehension of the Kinetics of Formation of Fe_3_O_4_ and Cofe_2_O_4_ NFs

**Figure 6:**
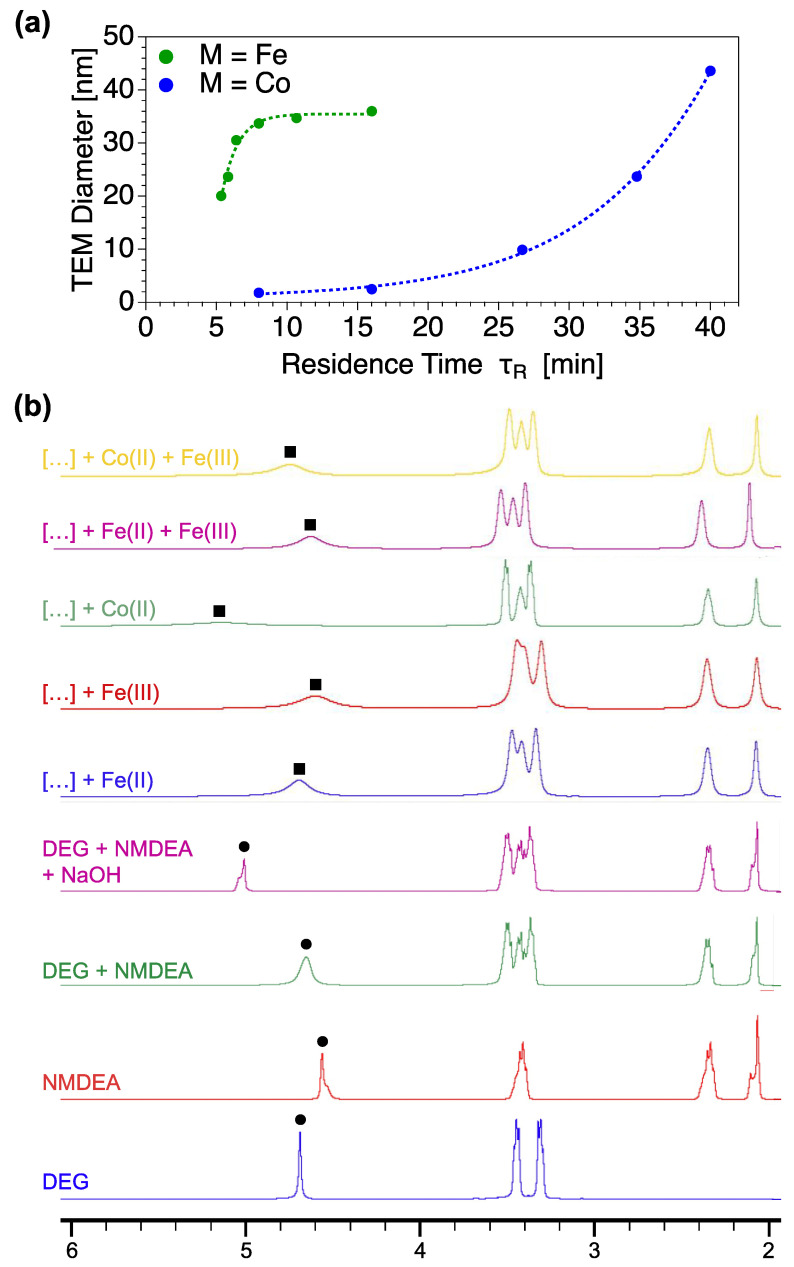
(**a**) Kinetic curves of NFs formation in the case of Fe_3_O_4_ (green) and CoFe_2_O_4_ (blue) and (**b**) ^1^H NMR spectra of different reactive media. In the spectra of the samples, “[…]” stands for “DEG + NMDEA + NaOH”.

As previously evidenced in Table 1, Fe_3_O_4_ and CoFe_2_O_4_ NPs present differences of size and shape for similar residence times. The evolution of the size from TEM as a function of the residence time is plotted in Figure 6a. It shows clearly that Fe_3_O_4_ and CoFe_2_O_4_ nanomaterials present different kinetics of growth. In order to understand such differences, ^1^H NMR experiments are carried out for different compositions of reactive media. Figure 6b presents the ^1^H NMR spectra of the solvents (DEG, NMDEA, DEG + NMDEA, DEG + NMDEA + NaOH) used as references and the spectra of the reactive media containing also one (Fe^II^, Fe^III^, Co^II^) or two (Fe^II^ + Fe^III^, Co^II^ + Fe^III^) of the metallic salt precursors. The main difference observed on all the spectra is located on the peak of alcohol functions of the solvents, marked by black circles for the references and by black squares for the samples. In all cases, the alcohol peak is shifted towards higher chemical displacement in presence of metallic salts when compared to the DEG + NMDEA reference spectrum, while the positions of the other peaks remain almost unchanged. This result strongly suggests that the preferential complexation site between metallic salts and the solvents is the alcohol functions, which are deprotonated in alcoholate functions due the presence of NaOH. More interestingly, it can be seen that Fe^II^, Fe^III^ and Co^II^ give rise to different modifications of the NMR spectral profile. Co^II^ is found to provoke a bigger shift of the alcohol peak with a huge enlargement of the full width at half maximum (FWHM) when compared to both Fe^II^ and Fe^III^. When focusing on the spectra of the reactive media containing the two metallic precursors (Fe^II^ + Fe^III^, Co^II^ + Fe^III^), it appears that the position and shape of the alcohol peak is closer to Fe^II^ and Fe^III^ than to Co^II^. Such result strongly suggests that the affinity between Fe^II^ or Fe^III^ and the solvents is higher than in the case of Co^II^. Briefly, when both iron and cobalt salts are in presence, the most stable complex is formed between iron salts and the solvents. This study of interactions at the molecular scale in the reactive media reveals a lack of complexation on Co^II^ sites. It may explain the slow formation of CoFe_2_O_4_ NFs when compared to Fe_3_O_4_ NFs. As a result, the kinetic of formation at the nano-scale [62] is governed by the molecular interactions and complexation competitions between the solvents and the cationic sites.

### 3.6. The Millifluidic Reactor as a Tool to Enhance Chemical Yield and Improve Reproducibility

**Figure 7:**
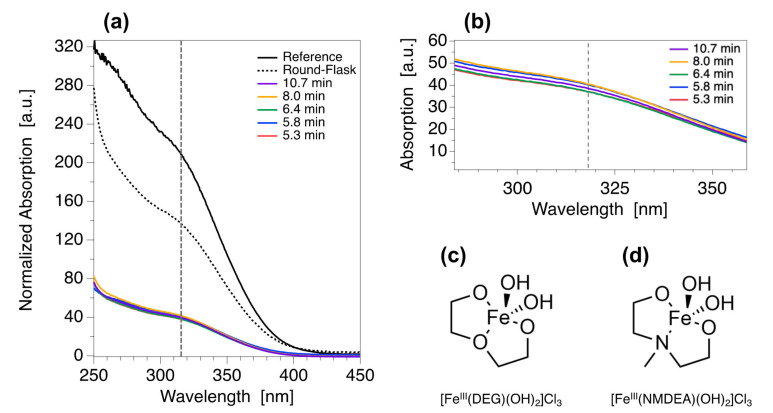
Measurements of the absolute chemical yield of the reaction producing magnetite nanoflowers. (**a**) Normalized absorption spectra of the supernatant of the crude reactive media, (**b**) zoom on the samples spectra in the region of interest and (**c**,**d**) molecular structures of the absorbing iron(III) complexes.

The yield of the reaction in NFs is evaluated in the case of Fe_3_O_4_ nanomaterials. The crude final media is analyzed by UV-Visible spectrophotometry in order to determine the absolute chemical yield of the reaction without considering the losses that may happen during the several washing steps. The NPs are separated by ultra-centrifugation to obtain a supernatant where only free iron(III) cations remain. The UV-Visible spectra of the initial reactive media (before reaction, reference) and the investigated samples are presented in Figure 7a. It can be seen that both the reference and the samples present similar spectral signature but with different intensities. All samples are found to be in the same range of absorption values as can be seen from the zoom in Figure 7b. A transition around 317 nm is evidenced and can be assigned to the absorption of complexes formed between free iron(III) and the polyol solvents (DEG, NMDEA) resulting in the formation of [Fe^III^(DEG)(OH)_2_]Cl_3_ and [Fe^III^(NMDEA)(OH)_2_]Cl_3_ shown in Figure 7c–d. The absolute chemical yield Φ is thus determined according to Equation 3:

(3) $$\phi =\frac{A_{ref}\left(317\right)-A_{sample}\left(317\right)}{A_{ref}\left(317\right)}$$

where *A_ref_*(317) is the absorbance value at 317 nm of the reference and *A_sample_*(317) is the absorbance value at 317 nm of the sample. All results are listed are listed in Table 2. Absolute chemical yields values are found to be independent from both flow rates and residence times with a mean obtained value of <Φ> = 80 ± 1% which is much higher when compared to chemical yields obtained for classical bulk syntheses that usually range from 30% to 40% (Figure 7a and Table 2, “round-flask” sample). Once again, these results can be explained by the efficient heat transfer inside the millifluidic reactor which enhances the reactivity of the chemical species. Production capacities are thus strongly increased by reducing iron losses, which also paves the way to a greener chemistry.

Another crucial point in nanomaterial sciences is the reproducibility of chemical synthesis, as properties of NPs are dictated by their physico-chemical features. In the case of Fe_3_O_4_ NFs, synthesis are reproduced 2 or 3 times for 6 different residences times ranging from 5.3 to 16 min. The as-synthetized NFs are analyzed by TEM to compare both mean diameters and polydispersity values. Table 3 presents the results obtained for each synthesis condition and both mean values and standard deviations on diameter (<*d*_0_>) and polydispersity (<*σ*>). A highly precise control over the shape on the resulting NFs is evidenced by the small standard deviations obtained for identical synthesis conditions. In all cases, the reproducibility in terms of mean diameter is greater than 95% (standard deviations inferior to 5%) and the control on polydispersity ranges around 90% (standard deviations of about 10%). As a result, the residence time is the main parameter governing the size distribution of the as-synthetized NFs and an excellent reproducibility is observed for syntheses performed in the same experimental conditions. The highly precise shape control allowed by the system is of great interest since the polyol routes hardly suffer from lack of reproducibility.

## 4. Conclusions

In this study, we showed the elaboration of a simple and safe multi-parametric millifluidic device for high temperature continuous-flow syntheses of iron oxide magnetic nanoclusters. The experimental setup showed excellent performance for the elaboration of ferrite NFs obtained by modified polyol route. We found that many physico-chemical features of the as-obtained NFs can be controlled by tuning the injection flow rate or the chemical composition of the initial reactive media. More precisely, a very good reproducibility was observed in terms of size and polydispersity of the NFs, which can be easily tuned by varying the residence time. Due to the homogeneity of the temperature in the reactor, the reactivity of the species was improved allowing a better control over the final chemical composition of the nanostructures and a high yield in nanomaterials production. Moreover, high injection flow rates up to 10 mL∙min^−1^ can be reached in this setup, which can allow easy and large scaling-up of the system to reach an industrial production of about 45 g of NFs per day. In addition to the elaboration of well-controlled nanostructures at a large scale, the millifluidic system may be used for time resolved studies of NPs formation (nucleation and growth) as shown in the case of cobalt ferrite. Finally, the multi-parametric system described herein is versatile and can be also extended to any other high-temperature synthesis so a great variety of nanomaterials could be synthetized.

## Figures and Tables

**Table 1:** Size distribution (*d*_0_, *σ*) and chemical composition (*R_F_*) of the as-synthetized NFs shown in Figure 5 for the different elements (Co, Fe, Mn) and residence times (*τ_R_*). Sample names correspond to the letters in Figure 5.

Element	Sample	*τ_R_* (min)	*d*_0_ (nm)	*σ*	*R*_0_	*R*_F_
Co	A–B	8	1.8	0.22	0.5	–
	C–D	16	2.5	0.23		–
	E	27	9.9	0.21		0.49 *
	F	35	23.7	0.26		0.46 *
	G	40	43.6	0.27		0.47 *
Fe	H	5.3	20.1	0.21	0.5	0.49 ^†^
	I	5.8	23.6	0.22		0.48 ^†^
	J	6.4	30.6	0.19		–
	K	8	33.7	0.24		0.49 ^†^
	L	11	35.2	0.22		0.47 ^†^
	M	16	36.2	0.24		–
Mn	N	2	60.3	0.22	0.25	0.23 *
	O	4	76.6	0.19		0.22 *
	P	8	87.5	0.26		0.22 *
	Q	16	104	0.23		0.23 *

* [Co]/[Fe] and [Mn]/[Fe] ratios are determined by AAS; see Table S1. ^†^ [Fe^II^]/[Fe^III^] ratios are determined by N analyses; see Figure S5 and Table S2.

**Table 2:** Values of absolute chemical yields *Φ* obtained in the case of Fe_3_O_4_ NFs shown in Figure 5. Sample names correspond to Figure 5 letters. “Round-Flask” stands for a classical synthesis of Fe_3_O_4_ NFs performed in a round-flask and is shown for comparison.

Sample	*τ_R_* (min)	*Q* (mL∙min^−1^)	*Φ* (%)
Round-Flask	60	–	34.2
H	5.3	3.0	81.7
I	5.8	2.75	80.2
J	6.4	2.5	81.8
K	8	2.0	80.1
L	11	1.5	81.0

**Table 3:** Diameters and polydispersity values obtained for syntheses performed in identical conditions for different residence times. Sample names correspond to Figure 5 letters and numbers in index indicate the number of repetitions. Mean values of diameter and polydispersity and their standard deviations are denoted as <*d*_0_> and <*σ*>, respectively. See Figure S6 for all size-distribution histograms.

*τ_R_* (min)	Sample	*d*_0_ (nm)	*σ*	<*d*_0_> (nm)	<*σ*>
5.3	H1	20.5	0.23	20.1 ± 0.9	0.21 ± 0.02
	H2	20.6	0.20		
	H3	19.1	0.20		
5.8	I1	22.9	0.21	23.6 ± 0.6	0.22 ± 0.01
	I2	23.8	0.23		
	I3	24.2	0.21		
6.4	J1	31.1	0.17	30.6 ± 0.7	0.19 ± 0.03
	J2	30.1	0.22		
8	K1	35.3	0.26	33.7 ± 1.6	0.24 ± 0.02
	K2	32.1	0.22		
11	L1	36.0	0.25	35.2 ± 0.8	0.22 ± 0.03
	L2	34.4	0.19		
16	M1	36.0	0.22	36.2 ± 0.2	0.24 ± 0.02
	M2	36.4	0.26		

## Data Availability

Not applicable.

## Supplementary Materials

The following are available online at https://www.mdpi.com/article/10.3390/nano12010119/s1. Equations S1–S6: expressions and descriptions of the equations used to make the temperature simulations inside the millifluidic channel; Figure S1: detailed on temperature simulations inside the millifluidic channel; Figure S2: size-distribution histograms of CoFe2O4 NFs obtained for different pressures; Figure S3: size-distribution histograms of Fe3O4 NFs obtained for different channel lengths and residence times; Figure S4: size-distribution histograms of CoFe2O4, Fe3O4 and MnFe2O4 obtained for different residence times; Figure S5: XANES analyses for Fe3O4 NFs stoichiometry determination; Figure S6: size-distribution histograms for reproducibility study on Fe3O4 NFs; Table S1: atomic absorption results for CoFe2O4 and MnFe2O4 NFs stoichiometry determination; Table S2: results of XANES analyses on Fe3O4 NFs.
