# Supplementary material for: High Temperature Continuous Flow Syntheses of Iron Oxide Nanoflowers Using the Polyol Route in a Multi-Parametric Millifluidic Device
Source: Nanomaterials (Basel). 2021 Dec 30;12(1):119. doi: 10.3390/nano12010119 (PMC8746638; doi:10.3390/nano12010119)
Supplement: Supplementary file 1 [file nanomaterials-12-00119-s001.zip › nanomaterials-1509166-supplementary.pdf]

SUPPLEMENTARY MATERIALS

High Temperature Continuous-Flow Syntheses of Iron Oxide Nanoflowers Using the Polyol Route in a Multi-Parametric Millifluidic Device

Enzo Bertuit[†], *Sophie Neveu*[†] *and Ali Abou-Hassan*[†,]*

[†]Sorbonne Université, UMR CNRS 8234, PHysico-chimie des Électrolytes et Nanosystèmes InterfaciauX (PHENIX), F-75005 Paris, France

[*]Author to whom correspondence should be addressed:

ali.abou_hassan@sorbonne-universite.fr

THEORETICAL BASIS

Heat transfer coefficient

The heat transfer coefficient *h* of the system can be expressed as follow:

$$h = \frac{2 \cdot \lambda \cdot Nu}{R_{in}} \qquad (S1)$$

where h is in W·m^{-2}·°C^{-1}, λ is the thermal conductivity of the mixture in W·m^{-1}°·C^{-1}, R_{in} is the inner radius of the tube in meters and Nu is the Nusselt number. The Nusselt number is a function of Helical (He) and Prandtl (Pr) dimensionless numbers, according to the empirical expression:

$$Nu = \left(\left(3.657 + \frac{4.343}{\left(1 + \frac{957}{Pr \cdot He^2}\right)^2} \right)^3 + \left(1{,}158 \cdot \left(\frac{He}{1 + \frac{0{,}477}{Pr}} \right)^{\frac{3}{2}} \right) \right)^{\frac{1}{3}} \qquad (S2)$$

Helical and Prandtl dimensionless numbers

The Helical number (He) can expressed as follow:

$$He = Re \cdot \sqrt{\frac{R_{in}}{R_C}} \qquad (S3)$$

where Re is the Reynolds number and R_C is the critical radius, that can be written as follows:

$$Re = \frac{v \cdot R_{in} \cdot \rho_{mix}}{2 \cdot \eta_{mix}} \qquad (S4)$$

$$R_C = R_{hel} + \frac{p}{2\pi} \qquad (S5)$$

with *v* the velocity of the fluid in m·s^{-1}, ρ_{mix} the density of the reactive mixture, η_{mix} the dynamic viscosity of the mixture in cP, R_{hel} the helical radius of the system in mm and *p* the pitch in mm between two adjacent spires. In our case, both density and dynamic viscosity are averaged considering the respective values associated to the two solvents (DEG and NMDEA, see Figure S1).

The Prandtl number (Pr) can be calculated as follow:

$$Pr = \frac{C_p \cdot \eta_{mix}}{1000 \cdot \lambda} \qquad (S6)$$

with C_p the specific heat of the solvent in kJ·kg^{-1}·°C^{-1}.

ADDITIONAL FIGURES

**Millifluidic channel characteristics**	
Tube inner diameter (mm)	**1,016**
Tube outer diameter (mm)	**1,5875**
Helical diameter (mm)	**29**
Flow rate (mL/min)	**0,5**
Pitch (mm)	**3**
**Linear flow velocity**	
Tube inner lenght (m)	20
Total inner volume (mL)	16,21463933
Volume per meter (mL/m)	0,810731967
Velocity (m/s)	0,010278777
**Reynold's Number**	
Dynamic viscosity DEG (cP)	35,7
Dynamic viscosity NDMEA (cP)	101
Density DEG	1,118
Density NMDEA	1,042
m DEG (g)	44,72
m NMDEA (g)	41,68
n DEG (mol)	0,42
n NMDEA (mol)	0,35
n% DEG	0,55
n% NMDEA	0,45
Dynamic viscosity mixture (cP)	65,32
Density mixture	1,08
Cinematic viscosity mixture (m2/s)	0,00006028
**Re =**	**0,173**
**Prandtl Number**	
Specific Heat DEG (kJ/kg/°C)	2,303
Thermal conductivity DEG (W/m/°C)	0,203
**Pr =**	**0,741**
**Helical Number**	
Tube inner radius (mm)	0,508
Helical radius (mm)	14,5
Pitch (mm)	3
Critical radius (mm)	14,97746483
**He =**	**0,032**
**Nusselt number & Heat transfert**	
**Nu =**	**3,7**
**h (W/m2/°C) =**	**730,7**
**Mass flow rate**	
mf (kg/s)	9,02941E-06

Figure S1. Details of the calculations made to obtain heat transfer coefficient of the system (denoted h, in $W{\cdot}m^{-1}{\cdot}{}^{\circ}C^{-1}$), in the specific case of a volumetric mixture of DEG and NMDEA. The mass flow rate depends on the flow rate (Q = 0.5 mL/min in the present example).

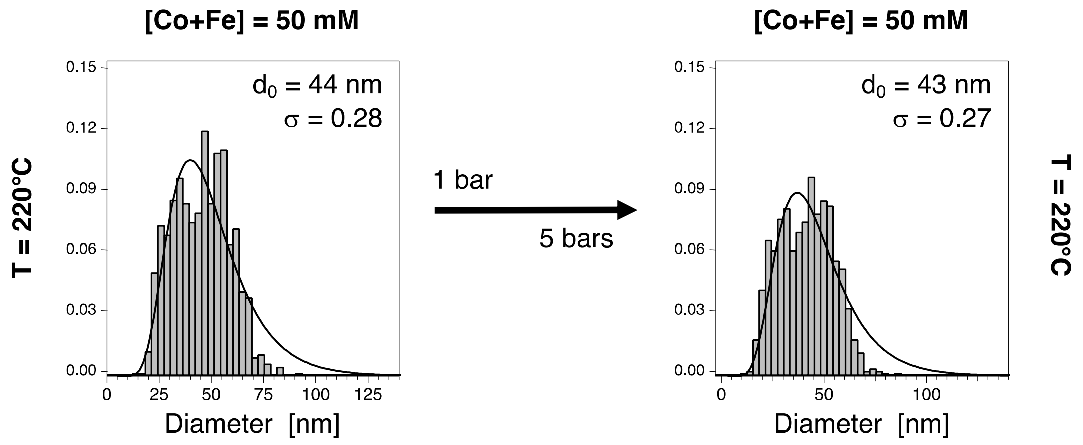

Figure S2. Size-distribution histograms obtained from TEM micrographs analyses in the case of $CoFe_2O_4$ NFs synthetized with an initial precursor concentration [Co+Fe] = 50 mM, at a temperature of 220°C and a pressure of 1 bar (left) or 5 bars (right). All Y-axis values represent the frequency, in percentage.

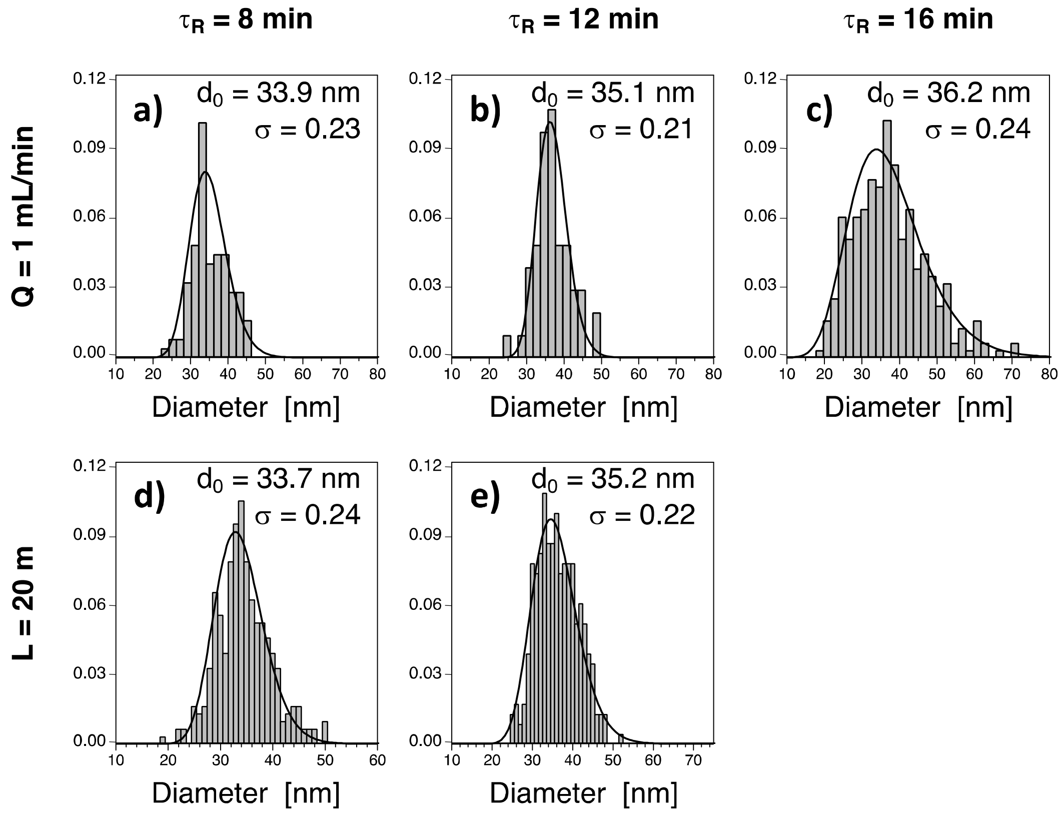

Figure S3. Size-distribution histograms obtained from TEM micrographs analyses in the case of Fe_3O_4 NFs synthetized for different flow rates and microfluidic channel lengths. a) Q = 1 mL/min, L = 10 m; b) Q = 1 mL/min; L = 15 m; c) Q = 1 mL/min, L = 20 m; d) Q = 2 mL/min; L = 20 m; e) Q = 1.4 mL/min, L = 20 m. All Y-axis values represent the frequency, in percentage.

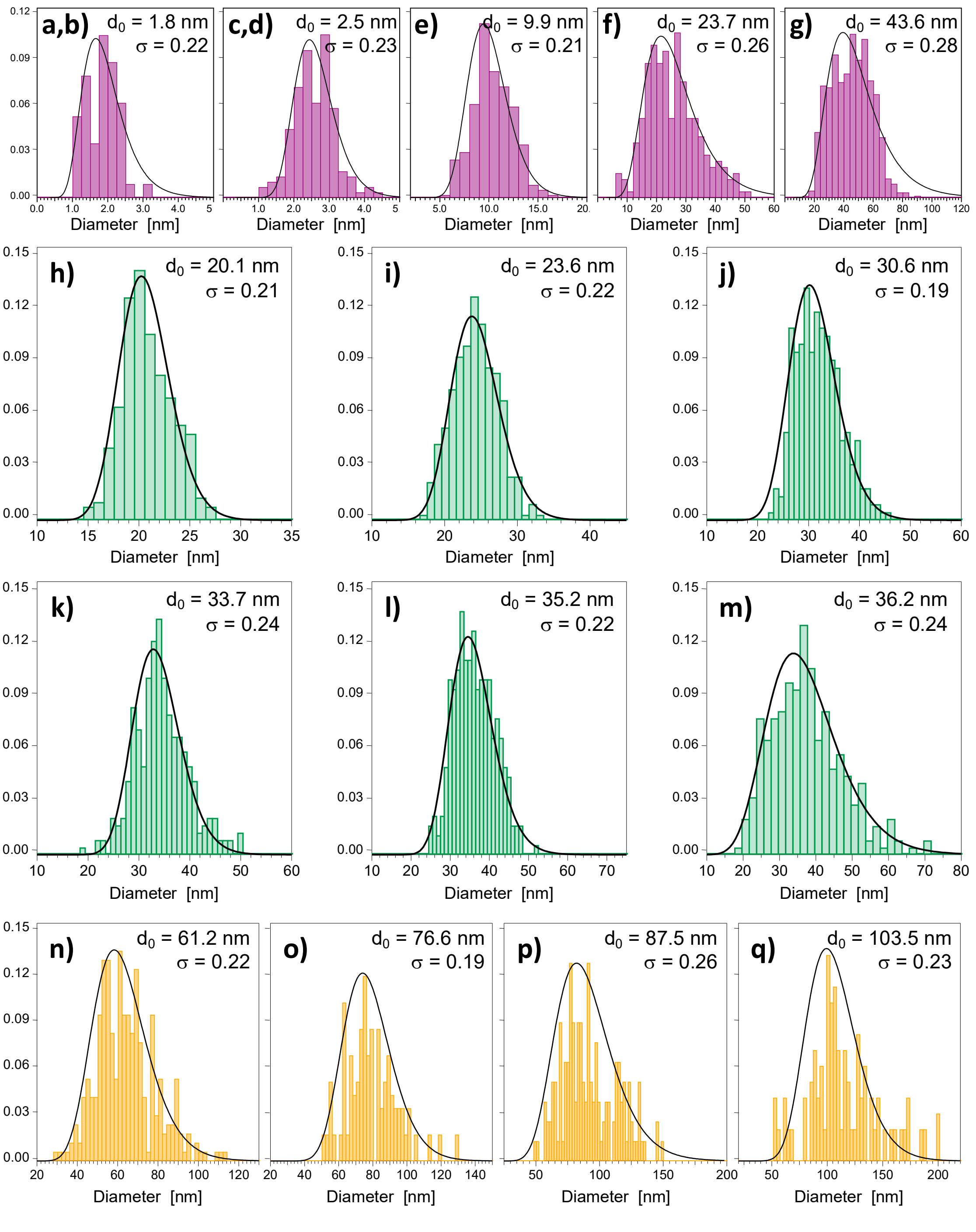

Figure S4. Size-distribution histograms obtained TEM micrographs analyses in the case of $CoFe_2O_4$ (purple), Fe_3O_4 (green) and $MnFe_2O_4$ (orange) for different residence times of a,b) 8 min; c,d) 16 min; e) 27 min; f) 40 min; g) 35 min; h) 5.3 min; i) 5.8 min; j) 6.4 min; k) 8 min; l) 11 min; m) 16 min; n) 2 min; o) 4 min; p) 8 min and q) 16 min. All Y-axis values represent the frequency, in percentage.

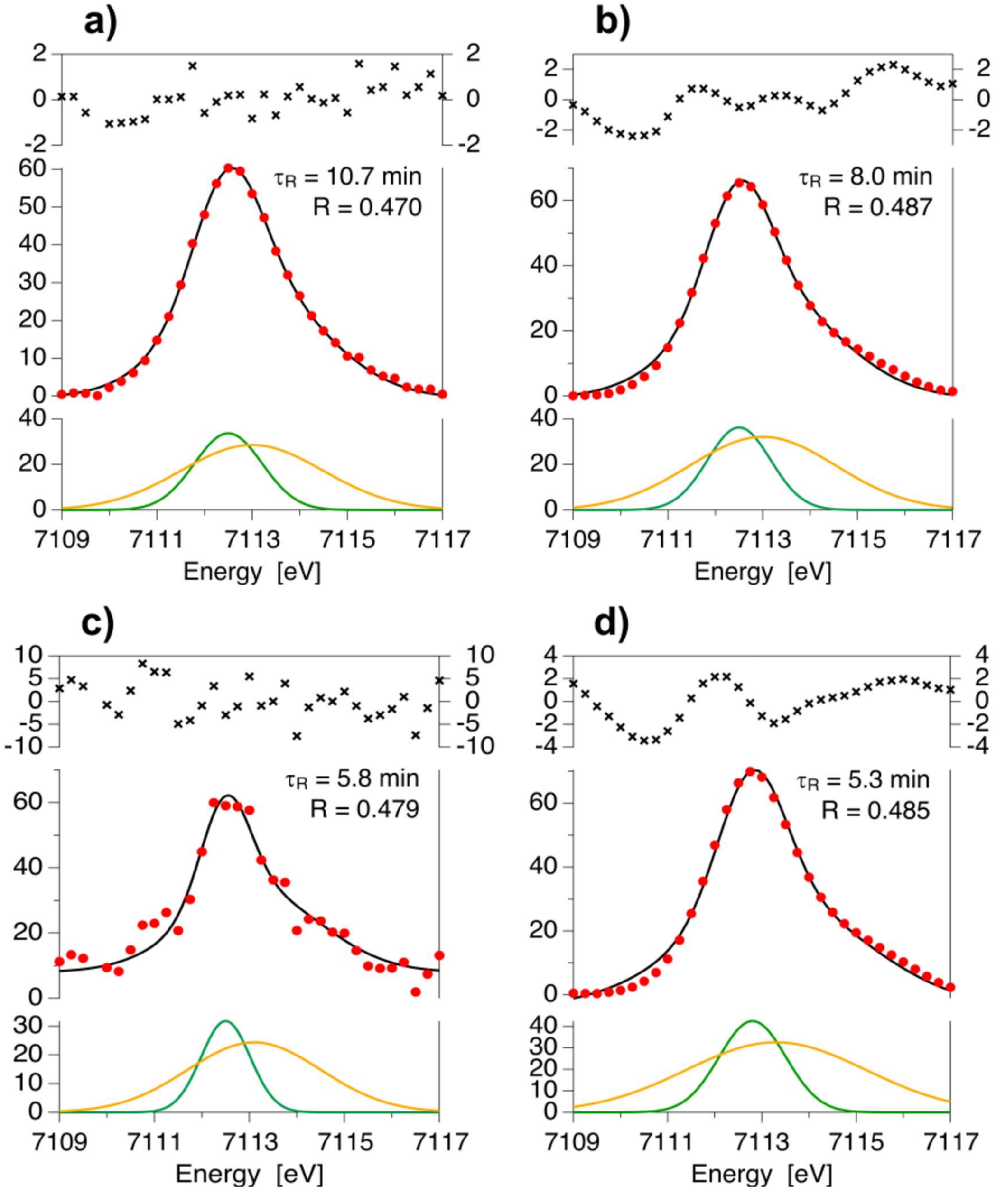

Figure S5. XANES analyses for Fe^{II}/Fe^{III} stoichiometry determination in the case of Fe_3O_4 NFs obtained for different residence times of a) 11 min; b) 8 min; c) 5.8 min and d) 5.3 min. Red points: pre-edge peak experimental data, black solid lines: 2–peaks models, black crosses: residuals, green solid lines: gaussian iron(II) contribution centered at 7112.5 eV, orange solid lines: gaussian iron(III) contribution centered at 7113 eV. See Table S2 for detailed results.

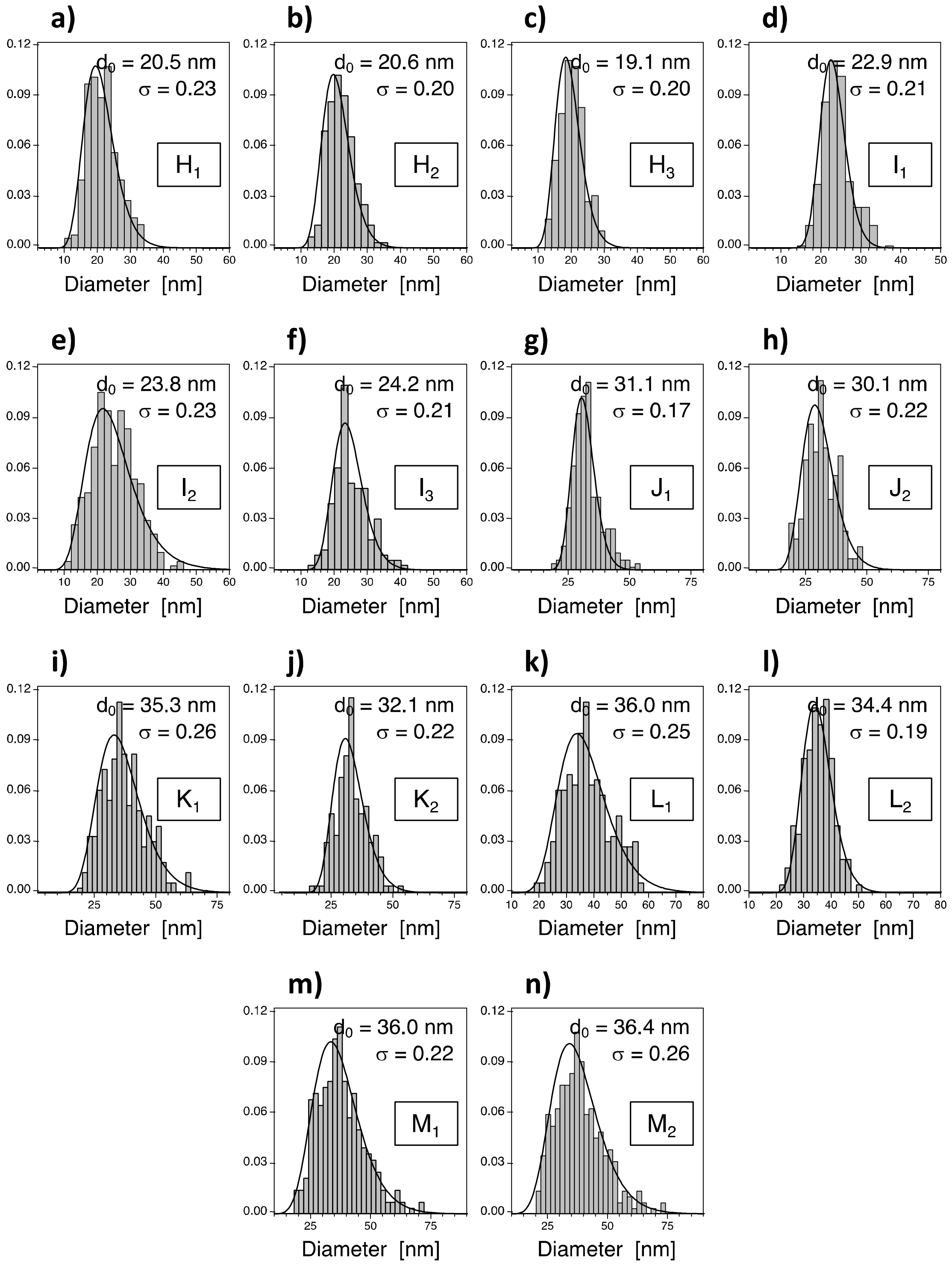

Figure S6. Size-distribution histograms obtained TEM micrographs analyses for reproducibility study on Fe_3O_4 NFs for different residence times of a–c) 5.3 min [samples H_1, H_2, H_3]; d–f) 5.8 min [samples I_1, I_2, I_3]; g,h) 6.4 min [samples J_1, J_2]; i,j) 8 min [samples K_1, K_2]; k,l) 11 min [samples L_1, L_2]; m,n) 16 min [samples M_1, M_2]. All Y-axis values represent the frequency, in percentage.

ADDITIONAL TABLES

Table S1. Determination of $[M^{2+}]/[Fe]$ ratios in the final NFs by atomic absorption spectroscopy, for M = Co and M = Mn.

M^{2+}	Sample	τ_R (min)	[Fe] mg/L	$[M^{2+}]$ mg/L	$R_F = [M^{2+}]/[Fe]$
Co	E	27	1.753	0.857	0.49
	F	35	0.771	0.351	0.46
	G	40	1.752	0.824	0.47
Mn	N	2	1.634	0.382	0.23
	O	4	1.091	0.237	0.22
	P	8	2.097	0.461	0.22
	Q	16	1.832	0.421	0.23

Table S2. Determination of $[Fe^{II}]/[Fe^{III}]$ ratios in the final NFs by XANES analyses, in the case of Fe_3O_4 NFs.

**τ_R (min)**	**Fe(III) contribution**		**Fe(II) contribution**		**R = Fe(II) / Fe(III)**	
	Area	Error	Area	Error	Value	Error
11	0.10665	0.0045	0.05008	0.0024	0.470	0.03
8.0	0.12404	0.0077	0.06038	0.0037	0.487	0.04
5.8	0.08588	0.0019	0.04114	0.0078	0.479	0.09
5.3	0.15555	0.0018	0.07538	0.0052	0.485	0.03
